# Effect of Qing'e Decoction on Leptin/Leptin Receptor and Bone Metabolism in Naturally Aging Rats

**DOI:** 10.1155/2020/2532081

**Published:** 2020-09-18

**Authors:** Pan Sun, Yuanyuan Zhang, Zhenpu Wei, Zhiqiang Wang, Shiming Guo, Yanping Lin

**Affiliations:** ^1^Academy of Integrative Medicine, Fujian University of Traditional Chinese Medicine, Fuzhou, Fujian 350122, China; ^2^Institute of Traditional Chinese Medicine, Fujian University of Traditional Chinese Medicine, Fuzhou, Fujian 350122, China; ^3^Institute of Acupuncture and Moxibustion, Fujian University of Traditional Chinese Medicine, Fuzhou, Fujian 350122, China; ^4^Zhangzhou Hospital of Traditional Chinese Medicine, Zhangzhou, Fujian 363000, China

## Abstract

Senile osteoporosis (SOP) is a common disease that has decreased bone strength as its main symptom. There is currently no medication that can treat SOP, and traditional Chinese medicine has advantages in slowing down bone aging. The present study aimed to observe the effects of Qing'e decoction on leptin, leptin receptor, sex hormone, and biochemical markers of bone metabolism in naturally aging rats and to explore its mechanism in regulating bone metabolism. The results revealed that, with the increase in age, the bone mineral density (BMD), bone strength, bone trabecula sparse, serum levels of leptin receptor (LEP-R), estradiol (E2), testosterone (T), core binding-factor *α*-1 (Cbf*α*-1), collagen-I (COL-I) and osteocalcin (OC), and the mRNA levels of leptin (LEP) and LEP-R in bone tissue decreased, while serum LEP levels increased in the female and male NS groups. The serum levels of LEP, LEP-R, E2, T, osteoprotegerin, Cbf*α*-1, COL-I, OC and bone alkaline phosphatase, and the mRNA levels of LEP and LEP-R in bone tissue in the female and male QED groups were higher than those in the same age and sex NS group, while the BMD, bone trabecular area percentage, maximum load, and maximum stress in the female and male QED groups were significantly higher than those in the same age and sex NS group. In conclusion, with the increase in age, the bone quality of naturally aging rats decreased gradually. Qing'e decoction can regulate the bone metabolism and increase the bone quality and delay bone aging, which may be achieved by increasing sex hormone, LEP, and LEP-R levels.

## 1. Introduction

Osteoporosis (OP), which is called a ‘silent killer', is a common degenerative disease characterized by reduced bone mass and bone microstructural changes, decreased bone strength, and increased risk of fracture. Senile osteoporosis (SOP) is a type of primary OP with a low conversion rate of bone metabolism [1]. With the increase in human life expectancy, the world is gradually entering an aging society, and the incidence and morbidity of OP are also increasing year by year [2]. However, there is currently a lack of an ideal treatment for SOP.

LEP, which is a peptide hormone synthesized and secreted by fat cells, is an endocrine protein mainly expressed in adipose tissue. In recent years, studies have shown that LEP plays a key role in regulating bone metabolism [3, 4]. It has been proved that leptin receptors are widely present in a variety of tissues, including bone tissue. Leptin receptors are expressed in bone marrow mesenchymal stem cells, osteoblasts, and other osteocytes [5, 6]. Studies on the pathogenesis of osteoporosis have found that an increase in adipocytes in the bone marrow can affect osteoblast differentiation and function, increase osteoclast activity, and influence bone mineralization, so leptin production by adipocytes may be an important regulator of the pathogenesis of osteoporosis [4]. However, its specific mechanism of action, especially in the aging process, needs further clarification.

Qing'e pills originated from *Prescriptions of the Bureau of Taiping People's Welfare Pharmacy* [7] and consist of *Eucommia ulmoides*, *Psoralea corylifolia L*, *Semen Juglandis*, and *Allium sativum L*. In the theory of Traditional Chinese Medicine, SOP is mainly caused by deficiency of spleen-kidney Yang, so tonifying the kidney and strengthening the spleen can treat SOP; Qing'e pills have the functions of invigorating the kidney and strengthening the spleen, bones, waist, and knee. Clinical research has confirmed that Qing'e pills have significant efficacy on SOP; Qing'e Pills can improve bone quality and bone density and relieve low back pain in different syndrome types of SOP [8, 9]. However, the mechanism of Qinge pills in the treatment of osteoporosis is still unclear. Therefore, this study intended to use aging rats as an SOP model to observe changes in LEP, leptin receptor (LEP-R) and serum bone metabolism markers with age, and the mechanism of action of the drug. Since rats cannot swallow pills, instead of Qing'e pills, oral decoction was used, which was called Qing'e decoction (QED).

## 2. Materials and Methods

### 2.1. Preparation of QED

According to the dosage of Chinese medicines in the 2000 edition of the Pharmacopoeia of the People's Republic of China (edited by the National Pharmacopoeia Commission) [10], the ratio of *Eucommia ulmoides*, *Psoralea corylifolia L*, *Semen Juglandis*, and *Allium sativum L* was 16 : 8 : 5 : 4. All herbs (from Good Agricultural Practice planting base) were purchased from the Chinese Medicine Hall of Fujian University of Traditional Chinese Medicine. All ingredients were identified by the Department of Pharmacy of the Second People's Hospital of Fujian Province to fulfil the quality requirements of the Pharmacopoeia of the People's Republic of China. The extract was also prepared by the aforementioned department. All the ingredients were soaked for 20 min, and the mixture was then decocted in boiling water for 45 min and vacuum-dried to yield a final concentration of 4.71 g crude drug/g. The conversion ratio between 200 g rats and 70 kg adults is 0.018, and the daily oral crude drug consumption for adults is 99 g; thus, the daily dose for rats was calculated to be 99 × 0.018/0.2 = ∼9 g/kg/day, which is equivalent to 1.91 g extract.

### 2.2. Animals

All animal experiments were conducted in strict accordance with the *Guide for the Care and Use of Laboratory Animals* and were approved by the Animal Care and Use Committee of Fujian University of Traditional Chinese Medicine (Approval No. 2020012). In total, 162 healthy, SPF-grade 6-month-old Sprague Dawley (SD) rats were purchased from Shanghai Slac Laboratory Animal Co., Ltd.(license no. SCXK (Shanghai) 2012–0002; certificate no. 2007000562039). SD rats were fed in the Experimental Animal Center of Fujian University of Traditional Chinese Medicine. The rats were fed with unified standard feed, which contained crude protein, crude fat, crude fiber, and other components. Each kilogram of feed contained 10–18 g calcium, 6–12 g phosphorus, and vitamin *D* ≥ 800IU. The feeding conditions of each group were the same, which would not affect the experimental results.

### 2.3. Experiment Design and Treatment

There were 81 male and 81 female SD rats of 6 months of age. A total of 9 male and female SD rats were randomly selected, and their tissues were collected after 1 week of adaptive feeding. In total, 36 rats (18 males and 18 females) were randomly selected from the remaining rats and divided into four groups: (i) female rats fed with QED (female QED group; *n* = 9); (ii) female rats fed with normal saline (female NS group; *n* = 9); (iii) male rats fed with QED (male QED group; *n* = 9); and (iv) male rats fed with normal saline (male NS group; *n* = 9). The remaining rats were fed normally and were grouped again at the age of 9, 12, and 15 months. The grouping method was the same as that described above. The gavage dose was calculated according to the body surface area, and the dose was adjusted weekly according to weight. Rats in the QED group were administered 1.91 g/kg/day QED extract dissolved with 2 ml normal saline. The saline group was administered normal saline (2 ml/day). After 3 months of continuous intervention, rats were euthanized with an overdose of pentobarbital (200 mg/kg), and their tissues were collected.

Blood was collected from the abdominal aorta, and the serum was centrifuged at 990 g for 15 min after standing for 4 h to separate the serum. The serum was separated and stored in the refrigerator at −80°C. The left tibia was separated, fixed in paraformaldehyde solution and EDTA decalcified. Next, Masson staining sections were prepared. The right tibia was separated for detection of bone density and bone biomechanics. The lumbars were subjected to LEP and LEP-R protein and mRNA detection.

### 2.4. Reagents

The following reagents were used in our study: RIPA protein lysate, protease inhibitor (Solarbio Science and Technology) (Beyotime Institute of Biotechnology); color prestained protein marker (Thermo Fisher Scientific, 26616); BCA protein concentration assay kit and TRIzol (Beyotime Institute of Biotechnology); estradiol (E2), testosterone (T), osteoporogeterin (OPG), receptor activator of nuclear factor-*κ*B ligand (RANKL), core-binding factor subunit *α*-1 (Cbf*α*-1), collagen-I (COL-I), osteocalcin (OC), bone alkaline phosphatase (BALP), and tartrate-resistant acid phosphatase 5b (TRACP-5b) ELISA kits (Wuhan Cusabio Bioengineering Co., Ltd.); reverse transcription kit and SYBR Premix Ex Taq^TM^ PCR Kit (Takara Bio, Inc.); PCR primers (Shanghai Bioengineering Technology Co., Ltd.); anti-LEP antibody (1 : 1000; Abcam, cat. no. ab3583); anti-LEP-R antibody (1 : 1000; Abcam, cat. no. ab104403); anti-*β*-actin antibody (1 : 1000; Cell Signaling Technology, Inc., cat. no. #4970); and anti-mouse IgG (H + L) (1 : 5000; Proteintech Group, Inc. cat. no. SA00001-1).

### 2.5. Measurement of Bone Mineral Density (BMD) and Bone Biomechanics

The BMD of the right tibia of rats was measured by a dual-energy X-ray 4500W BMD instrument. The maximum load (KN) and the maximum stress (n/mm^2^) were measured in an ig-a1000n universal material testing machine.

### 2.6. Morphology

The metaphyses of the left proximal tibia of the rats in each group were isolated and fixed with 4% paraformaldehyde solution for 48 h. Then, they were decalcified in EDTA-decalcified solution for 90 days and embedded in paraffin. A tissue slicer was used to prepare slices of 5 mm thickness along the longitudinal section of the long axis of the tibia. After Masson staining, changes in morphology and structure of the metaphyses of the proximal tibia (excluding tibial plateau) were observed with a microscope. The percentage of trabecular area (trabecular area in visual field/total area measured) was measured using a Motic Med 6.0 digital medical image analysis system (Motic Instruments).

### 2.7. ELISA

Frozen serum was incubated at room temperature for 30 min. Then, 100 *μ*l standard at different concentrations and serum were added to the well. Then, 50 *μ*l enzyme-linked affinity was added to each well and incubated at 37°C for 60 min. Next, the solution was removed from inside the well and washed thoroughly. Then, substrates I and II were added to the wells and mixed and allowed to react for 15 min at room temperature in the dark. The reaction was stopped, and 30 mins later, the optical density (OD) of each well was measured using a microplate reader set to 450 nm. A standard curve was created to calculate the LEP, LEP-R, E2, T, BALP, TRACP-5b, OC, Cbf*α*-1, OPG, and RANKL contents in serum.

### 2.8. Reverse Transcription-Quantitative PCR (RT-qPCR)

Total RNA was extracted from the tissue according to the TRIzol method, and the RNA concentration and OD were measured with a UV spectrophotometer. An OD260/OD280 between 1.8 and 2.0 indicated that the RNA concentration was appropriate. A total of 1 *μ*g RNA was used for the reaction, alongside 4 *μ*l 5X PrimeScript^TM^ Buffer (for Real Time), 1 *μ*l PrimeScript^TM^ RT Enzyme Mix 1, 1 *μ*l Oligo dT Primer, 1 *μ*l/concentration total RNA, and DECP water (20 *μ*l). RT was performed on a PCR instrument, and cDNA was obtained for PCR. The primers used were as follows: LEP primer (F) 5′-TCACACACGCAGTCGGTATC-3′ and reverse (R) 5′-GAGGAGGTCTCGCAGGTTCT-3′; LEP-R F 5′-TTGCGTATGGAAGTCACAGATG-3′ and R 5′-CACCTGGACCTCGTATGAAGAC-3′; and GAPDH F 5′-GGCACAGTCAAGGCTGAGAATG-3′ and R 5′-ATGGTGGTGAAGACGCCAGTA-3′.

According to SYBR GREEN instructions, the reaction system was set up as follows: SYBR Premix Ex Taq^TM^ II (2X), 10 *μ*l; ROX Reference Dye II (50X), 0.4 *μ*l; F primer (10 *μ*mol/l), 0.8 *μ*l; R primer (10 *μ*mol/l), 0.8 *μ*l; 2 *μ*l cDNA template; and 6 *μ*l DECP water. The reaction conditions were as follows: Predenaturation at 95°C for 30 sec, denaturation at 95°C for 5 sec, annealing and extension at 60°C for 34 sec (40 cycles to obtain the amplification curve and Cq value), and 95°C for 30 sec, 95°C for 3 sec, and 60°C for 30 sec (40 cycles to obtain the dissolution curve).

### 2.9. Western Blotting

After extracting total protein from bone tissue, the BCA method was used for protein quantification. Gels were prepared according to the instructions of the SDS-PAGE kit (Solarbio Science & Technology, P1200) and 5 *μ*l marker, and 40 *μ*g protein samples were loaded. The initial voltage was 20 V for 10 min, 80 V for 30 min, and 120 V for 90 min. After electrophoresis, the proteins were transferred to a PVDF membrane, and membranes were blocked by 5% nonfat milk for 2 h and incubated with the primary antibodies overnight at 4°C. TBST was used to wash the membranes, and the goat peroxidase-conjugated secondary antibody was incubated at room temperature for 1 h before chemiluminescence imaging.

### 2.10. Statistical Analysis

Statistical analysis was performed using SPSS software (version 23.0; IBM Corp.). Data were analyzed using one-way ANOVA with the post hoc Tukey's test was used to compare multiple groups. Data are presented as the mean ± standard deviation. *P* < 0.05 was considered to indicate a statistically significant difference. The correlation between LEP, LEP-R, BMD, E2, T, OPG, RANKL, Cbf*α*-1, COL-I, OC, BALP, and TRACP-5b was analyzed by bivariate correlation test.

## 3. Results

### 3.1. QED Can Improve the Bone Quality of Aging Rats

**Figure 1:**
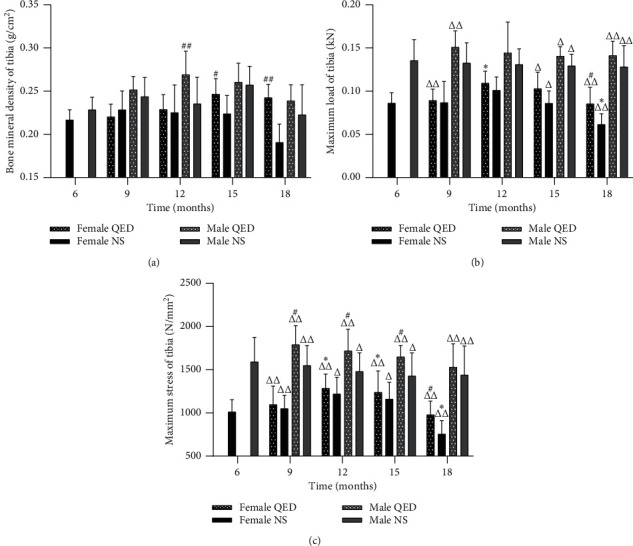
Qing'e decoction can improve the bone quality of aging rats. (a) Bone mineral density of tibia. (b) Maximum load of tibia. (c) Maximum stress of tibia. ^*∗*^*P* < 0.05, ^*∗∗*^*P* < 0.01 vs. same-sex 6-month-old group; ^*#*^*P* < 0.05, ^*##*^*P* < 0.01 vs. same-age and sex normal saline group but different intervention group; ^ΔΔ^*P* < 0.05, ^ΔΔ^*P* < 0.01 vs. same-intervention and age group but different sex.

The bone density of male rats reached the highest peak at 15 months of age, while that of female rats reached the highest peak at 12 months of age and then it gradually decreased. The decrease in bone density in the QED group was slower than that in the NS group (Figure 1(a)). The maximum load and stress of tibia of male and female rats decreased gradually after 12 months of age. The rate of decrease in the QED group was slower than that in the NS group (Figures 1(b) and 1(c)). The results showed that the loss of bone mass and the decrease in bone strength occurred after 12 months of age. The bone density and bone strength of rats in QED group were higher than those of the NS group. This indicates that QED can slow down the loss of bone mass, improve the bone quality, and delay the aging of bones.

### 3.2. Morphology

**Figure 2:**
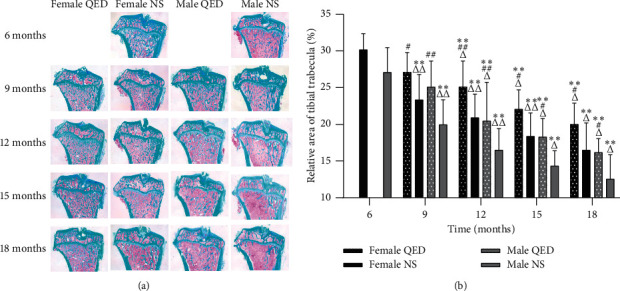
QED can increase the trabecular bone area. (a) Compared with that in the same-sex NS group of the same age, the bone trabeculae in the QED group had better morphological and structural integrity, higher number, and smaller spacing and less fracture phenomenon (magnification, x20). (b) Measurement results of trabecular area (%) of tibia in each group. ^*∗*^*P* < 0.05, ^*∗∗*^*P* < 0.01 vs. same-sex 6-month-old rats; ^*#*^*P* < 0.05, ^*##*^*P* < 0.01 vs. same-age and sex NS group but different intervention group; ^Δ^*P* < 0.05, ^ΔΔ^*P* < 0.01 vs. same-intervention and age group but different sex. QED, Qing'e decoction; NS, normal saline.

In this experiment, the bone trabeculae of the upper tibia were stained with Masson staining after conventional paraffin-embedded sectioning. The bone trabeculae were colored blue/green, and the bone marrow cells were red. The results showed that the bone trabeculae of the upper tibia of 6-month-old rats were arranged regularly and closely, distributed evenly, connected in a network shape, and continuous without fracture and had small interstices. With the increase in age, the number of bone trabeculae decreased gradually; the morphology and structure became sparse and thin; the distribution of trabeculae was scattered and irregular; the coincidence between trabeculae decreased; the porosity increased; the spacing increased; and there were fractures. Compared with that of the NS group of the same age and sex, the bone trabeculae in the QED group had better morphological and structural integrity, higher number, smaller spacing, and less fractures. The results showed that, with the increase in age, the percentage of trabecular area in the female and male groups decreased gradually, which was significantly different from that observed in the same sex groups (*P* < 0.01). Compared with the same-sex NS group, the percentage of bone trabecular area in both the female and male QED groups increased significantly (*P* < 0.05 and *P* < 0.01, respectively). In the same age and intervention group, the male was higher than the female in different genders. There were significant differences between the 12-, 15-, and 18-month-old QED group and the NS group (*P* < 0.05 and *P* < 0.01) (Figures 2(a) and 2(b)).

### 3.3. Effect of QED on Serum LEP, LEP-R, and Bone Metabolism Biomarkers

**Figure 3:**
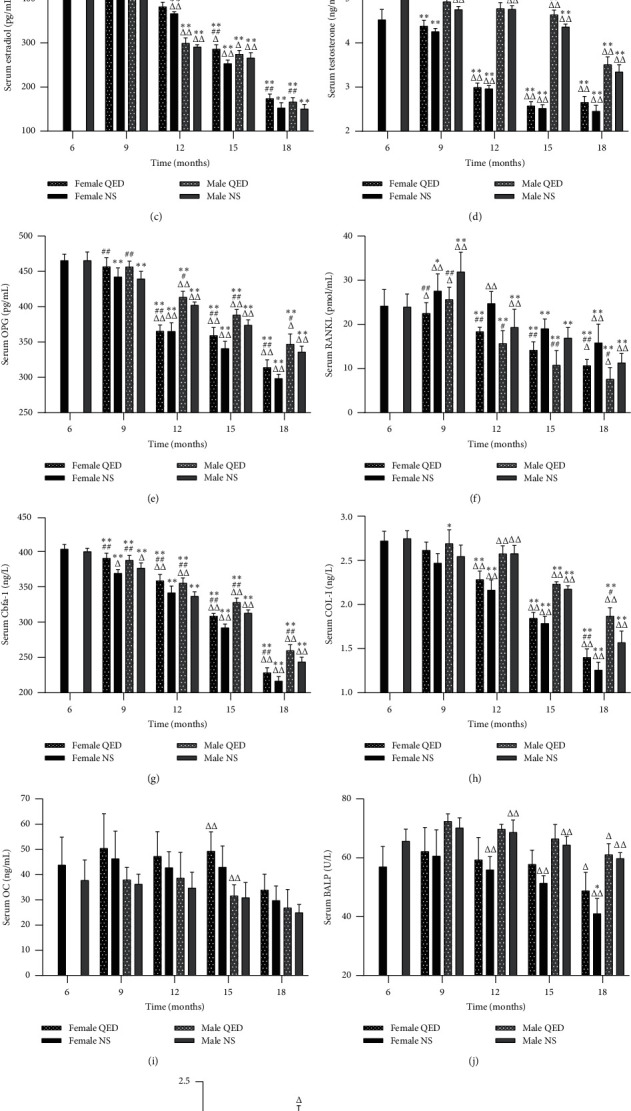
Qing'e decoction can affect serum leptin, leptin receptor, and bone metabolism markers. These biomarkers were detected by ELISA. ^*∗*^*P* < 0.05, ^*∗∗*^*P* < 0.01 vs. same-sex 6-month-old rats; *P* < 0.05, ^*##*^*P* < 0.01 vs. same-age and sex NS group but different intervention group; ^Δ^*P* < 0.05, ^ΔΔ^*P* < 0.01 vs. same-intervention and age group but different sex. OPG, osteoprotegerin; RANKL, receptor activator of nuclear factor-*κ*B ligand; Cbf*α*-1, core-binding factor subunit *α*-1; COL-I, collagen-I; OC, osteocalcin; BALP, bone alkaline phosphatase; TRACP-5b, tartrate-resistant acid phosphatase 5b.

The serum LEP levels in the female and male NS groups gradually increased with age, while the serum LEP-R levels exhibited the opposite trend. Serum LEP and LEP-R levels in the female and male QED groups were higher than those in the same age and sex NS groups, but there was no significant difference. The levels of serum LEP and LEP-R in males were higher than those in females in the same age and the same-intervention group. The male 15-month-old QED group and the 12- and 15-month-old NS groups were significantly different from the female group (*P* < 0.05, *P* < 0.01) (Figures 3(a) and 3(b)).

With the increase in age, the serum E2 and T levels in both the female and male NS groups decreased significantly (*P* < 0.05). The E2 levels of the female QED groups were higher than those of the NS groups of the same sex and age (*P* < 0.05 and *P* < 0.01, respectively). The T levels of the male QED groups at 9 and 15 months of age were higher than those of the NS groups at the same sex and age (*P* < 0.05 or *P* < 0.01) (Figures 3(c) and 3(d)).

Serum OPG and RANKL levels were significantly increased from 6 to 9 months in both the male and female NS groups (*P* < 0.01) and were significantly decreased from 9 to 18 months of age (*P* < 0.05 or *P* < 0.01). Compared with those of the NS groups of the same age and sex, the serum OPG levels were increased, while the RANKL levels were decreased in the female and male QED groups (*P* < 0.05 or *P* < 0.01). The serum OPG levels in males were higher than those in females, while the serum RANKL levels were lower (Figures 3(e) and 3(f)).

The serum Cbf*α*-1, COL-I, and OC levels of the female and male NS groups decreased gradually, and the Cbf*α*-1 and COL-I levels were significantly different from the 6 months of the same gender (*P* < 0.05 or *P* < 0.01). However, OC levels did not differ significantly. The serum levels of Cbf*α*-1, COL-I and OC in the female and male QED groups were higher than those in the same-age NS group, and Cbf*α*-1 was significantly different in QED groups regardless of age (*P* < 0.01). COL-I exhibited significant differences in the 18-month-old QED groups (*P* < 0.05), whereas there was no significant difference in OC. Compared with those in the different sex and the same intervention group of the same age, the serum COL-I levels in the male groups were higher than those in the female groups, while the opposite was observed for OC. Serum Cbf*α*-1 in the 6- to 12-month-old male groups was higher than that in the female groups, while it was lower in the 12- to 15-month-old male groups, and had significant differences at each age (*P* < 0.05 or *P* < 0.01). The serum COL-I of the 12-, 15-, and 18-month-old QED groups were significantly different from those of the NS groups (*P* < 0.01). OC was significantly different only in the 15-month-old QED group (*P* < 0.01) (Figures 3(g) and 3(i)).

The serum BALP level of the female and male NS groups increased from 6 to 9 months, while it decreased from 9 to 18 months. The serum TRACP-5b level decreased with age. BALP and TRACP-5b in female and male QED groups were higher than those in the same-sex NS groups. The serum BALP and TRACP-5b levels in the male groups were higher than those in the female groups, and BALP was significantly different in the 18-month-old QED and the 12-, 15-, and 18-month-old NS groups (*P* < 0.01), while TRACP-5b was significantly different in the 9-month-old QED and NS groups (*P* < 0.05 and *P* < 0.01) (Figures 3(j) and 3(k)).

### 3.4. LEP and LEP-R mRNA and Protein Expression

**Figure 4:**
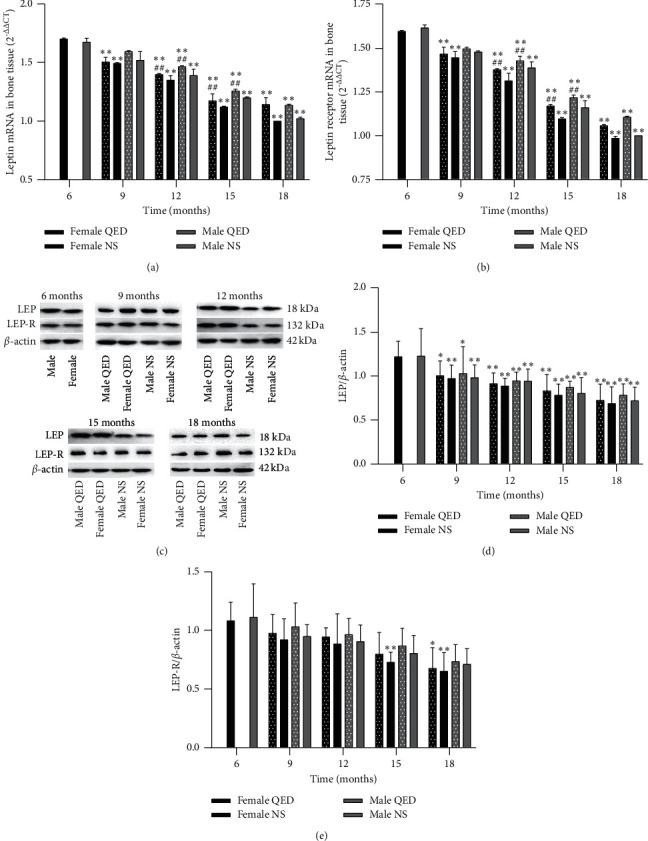
Effect of Qing'e decoction on bone tissue LEP and LEP-R mRNA and protein expression. Results of reverse transcription-quantitative PCR indicating the relative mRNA expression levels of (a) LEP and (b) LEP-R. (c) Images of protein expression analyzed by western blot assay. Relative protein expression levels of (d) LEP and (e) LEP-R. ^*∗*^*P* < 0.05, ^*∗∗*^*P* < 0.01 vs. same-age 6-month-old rats; ^*#*^*P* < 0.05, ^*##*^*P* < 0.01 vs. same-age and sex normal saline group but different intervention group. LEP, leptin; LEP-R, leptin receptor.

With the increase in age, the expression level and protein content of LEP and LEP-R mRNA in bone tissue of the male and female NS groups decreased gradually. The expression of LEP and LEP-R mRNA and protein in the same-age and sex groups was higher than that in the NS group, but there was no significant difference. No significant differences were found males and females of the same-age and intervention groups (Figures 4(a)–4(e)).

### 3.5. Correlation between Serum LEP, LEP-R, BMD, and Other Serum Bone Metabolic Markers

The correlation between LEP, LEP-R, BMD, E2, T, OPG, RANKL, Cbf*α*-1, COL-I, OC, BALP, and TRACP-5b was analyzed by the bivariate correlation test, with Pearson as the correlation coefficient. The results showed that serum LEP was negatively correlated with E2, T, OPG, RANKL, Cbf*α*-1, COL-I, and OC but not with BMD, serum BALP, or TRACP-5b. Serum LEP-R was positively correlated with, E2, T, OPG, RANKL, Cbf*α*-1, COL-I, OC, BALP, and TRACP-5b but not with BMD (Table 1).

## 4. Discussion

The bone remodeling process of normal people is maintained by the ‘bone formation and resorption' coupling mechanism. As age increases, the uncoupling of bone formation and resorption is the main cause of bone loss. The results of BMD, biomechanics (Figures 1(a)–1(c)), and bone histomorphology (Figures 2(a) and 2(b)) showed that age-related bone loss begins to appear in female rats at 12 months of age and in male rats at 15 months of age, and the age-related bone loss in male rats is relatively slow, while the decrease in BMD is later and slower than in female rats. The reduction in bone metabolism leads to bone formation and bone resorption uncoupling, and osteoblast cell function is decreased while osteoclast functional activity is enhanced, eventually resulting in bone resorption greater than bone formation; thus, bone density is reduced, thereby causing bone aging. The morphological changes and biochemical markers of naturally aging rats at 18 months of age were similar to those of OP, which established a theoretical basis for further research in this subject.

Multiple factors are involved in the bone remodeling process, and LEP is one of them. Previous studies have shown that serum LEP levels increase with age in normal rats, while LEP-R levels decrease with age [11]. The soluble LEP-R is the major conjugate of LEP in the peripheral circulation and is an important factor in determining the total amount of LEP in the peripheral circulation. It can be used as a measure of the total quantity of bioactive LEP [12, 13]. Excessive LEP cannot play a role in inhibiting fat synthesis, indicating that there may be a ‘LEP resistance' effect during aging, and this effect gradually increases with age [14]. Recent studies have shown that LEP plays a critical role in regulating bone metabolism [3, 4]. However, since the mechanism of the effect of LEP on bone metabolism involves numerous aspects, there is no convincing conclusion.

With the increase in age, the serum LEP levels of the female and male NS groups gradually increased, whereas the serum LEP-R levels decreased. The male serum LEP and LEP-R levels were higher in the same-intervention group than in the same-age group (Figures 3(a) and 3(b)). In bone tissue, we can see a gradual decrease in LEP mRNA and protein levels (Figures 4(a)–4(e)). As the expression of LEP-R decreased with age, the total LEP in serum increased but did not play a role with increasing age, probably due to “LEP resistance”. The serum test results of bone metabolism-related biomarkers showed that serum E2, T, OPG, RANKL, Cbf*α*-1, COL-I, OC, and BALP levels in the female and male NS groups decreased significantly with age, while TRACP-5b significantly increased. There was a significant difference between the age of each month and the age of 6 months (*P* < 0.05 or *P* < 0.01) (Figures 3(a)–3(k)). In this experiment, the bivariate correlation test was used to analyze the correlation between biomarkers. The results showed that serum LEP was negatively correlated with E2, T, OPG, Cbf*α*-1, COL-I, and BALP, while serum LEP-R was positively correlated with the levels of E2, T, OPG, RANKL, Cbf*α*-1, COL-I, OC, BALP, and TRACP-5b, indicating that serum LEP and LEP-R were closely associated with bone metabolism. In addition, the secretion of sex hormones decreased with age, and E2 and T were positively correlated with LEP-R but negatively correlated with LEP, indicating that the secretion of sex hormones was closely associated with the expression of LEP and LEP-R (Table 1).

OP is a disease of “guku,” “guwei,” and “gubi” in Traditional Chinese Medicine, mostly a spleen-kidney yang deficiency syndrome. In the theory of Traditional Chinese Medicine, the kidney includes the endocrine, urinary and reproductive systems, while the spleen includes the digestive system. Traditional Chinese Medicine considers that the kidney is involved in generating marrow and dominating bone, which means that the kidney plays an important role in the growth and development of bones. As age increases, kidney essence deficient, cannot generate marrow to raise bone, which leads to atrophic debility of the bone, namely OP. Therefore, the clinical treatment methods mostly rely on tonifying the kidney and strengthening the spleen. QED consists of *Eucommia* (fried salt), *Psoralea corylifolia L* (fried salt), *Semen Juglandis* (fried), and *Allium sativum L*. Decocting these four herbs together helps to warming up the yang, tonifying the kidney, warming up the middle, and strengthening the spleen, muscles, and bones. Previous studies have shown that QED can significantly regulate the bone metabolism-related factors, promote the bone formation, inhibit the bone absorption, and play an active role in the prevention and treatment of OP [15–18].

Our results showed that the serum LEP level increased and the serum LEP-R level decreased with age. In serum and bone tissue, LEP and LEP-R levels of the female and male QED groups were higher than those of the same-sex NS group, although the difference was not significant (Figures 3(a) and 3(b), (Figures 4(a)–4(e)). The serum LEP and LEP-R levels were higher in males than in females. Compared with the findings in the same-sex NS group of the same age, the shape and structure of bone trabeculae in the QED group were more complete, higher in number and smaller in spacing, and exhibited less fractures. The BMD, percentage of trabecular area, maximum load, and maximum stress in the female and male QED groups were higher than those in the same-age group. This result suggests that, with the increase in age, LEP may affect the bone metabolism, improve the bone quality, and delay the bone aging in various ways.

Some studies have shown that LEP can significantly increase the expression of Cbf*α*-1, COL-I, OC, and BALP [19, 20], which play key roles in the proliferation, differentiation, and maturation of osteoblasts [21–27], indicating that LEP can enhance bone formation. The experimental results showed that, with the increase in age, the levels of Cbf*α*-l, COL-I, and OC in the serum of female and male QED groups were significantly higher than those of the same-sex NS groups, and there were significant differences in Cbf*α*-1 at different ages (*P* < 0.01). COL-I levels exhibited a significant difference at 18 months of age (*P* < 0.05 or *P* < 0.01), but there was no significant difference in OC levels, while serum LEP-R levels were positively correlated with Cbf*α*-1, COL-I, and OC. Furthermore, the serum BALP levels in the female and male QED groups were higher than those in the same-sex NS group. These findings suggested that QED can increase the concentration of Cbf*α*-l in serum and then increase the concentration of COL-I, OC, and BALP, which may be caused by the increase in LEP-R in serum, which promotes the differentiation and maturation of osteoblasts, increases the functional activity of osteoblasts, and adjusts the uncoupling of bone absorption and bone formation caused by aging, thus effectively preventing and treating primary OP (Figures 3(g)–3(j)).

LEP can inhibit the differentiation, development, maturation, and function of osteoclasts through the regulation of RANKL/RANK/OPG, a signaling pathway closely associated the with osteoclast functional activity [28–30]. The ratio of OPG to RANKL is negatively correlated with osteoclast differentiation [31]. LEP can increase the expression of OPG in osteoblasts and bone marrow stromal cells and inhibit the expression of RANKL, thus inhibiting the differentiation of osteoclasts, reducing the generation of osteoclasts, and inhibiting the bone absorption [32]. The results showed that, with the increase in age, the levels of serum OPG and RANKL in the female and male QED groups were significantly higher than those in the same-sex NS group (*P* < 0.05 or *P* < 0.01). It has been suggested that QED can increase the expression of OPG, enhance the competition with RANKL, reduce the concentration of RANKL, and decrease the binding rate of RANKL and RANKL, which may be associated with the increase in expression of LEP-R and the upregulation of the expression of LEP with biological activity, which increases the ratio of OPG/RANKL, inhibits the differentiation and maturation of osteoclasts, reduces the expression of Tracp-5b (an effective marker of osteoclast number and functional activity), and inhibits bone resorption and delays bone aging (Figures 3(e), 3(f) and 3(k)).

In the aging process, an important feature is the decrease in gonadal function. There is a close association between sex hormones and LEP. Some studies have shown that the expression level of sex hormones increases with the increase in LEP, and there is a positive correlation between the two [33, 34]. LEP can increase the estrogen concentration by increasing aromatase activity through LEP-R and STAT3, thus promoting bone formation. Moreover, estrogen can affect STAT3 phosphorylation levels through binding to the estrogen receptor and then acting on LEP and LEP-R [35, 36]. In conclusion, LEP is closely associated with sex hormones. LEP can regulate bone metabolism by affecting the expression of sex hormones. The results showed that, with the increase in age, the serum E2 and T levels of the female and male QED groups were significantly higher than those of the same-sex NS groups, and the E2 levels of the female and male QED groups were significantly higher than those of males of 18 months of age, while the T levels of males at 9 and 15 months of age were significantly different (*P* < 0.05 or *P* < 0.01). This suggested that serum E2 and T levels in the female and male NS groups decreased significantly with increasing age, while QED could increase the expression of E2 and T by upregulating the expression of LEP-R, which may involve proteins of the LEP pathway such as STAT3, thus regulating bone metabolism and adjusting the uncoupling of bone absorption and bone formation due to aging in order to effectively prevent and treat primary OP (Figures 3(c) and 3(d)).

## 5. Conclusion

With the increase in age, the level of serum sex hormones decreased and the expression of serum LEP increased and the expression of LEP-R decreased, which led to the decrease of the OPG/RANKL ratio, disorder of bone metabolism, greater bone absorption than bone formation, and decrease in bone quality.

QED can regulate the bone metabolism, improve the bone quality, delay the bone aging, and prevent the and cure primary OP. This effect may be achieved by increasing the level of sex hormones, the expression of LEP and LEP-R, and the OPG/RANKL ratio.

## Figures and Tables

**Table 1:** Correlation analysis of LEP, LEP-R, and bone metabolism markers.

	BMD	E2	T	OPG	RANKL	Cbfa1	Col-I	OC	BALP	TRACP-5b
LEP	0.136	−0.480^*∗∗*^	−0.170^*∗*^	−0.405^*∗∗*^	−0.442^*∗∗*^	−0.407^*∗∗*^	−0.330^*∗∗*^	−0.198^*∗*^	−0.095	−0.058
LEP-R	0.132	0.744^*∗∗*^	0.738^*∗∗*^	0.840^*∗∗*^	0.540^*∗∗*^	0.793^*∗∗*^	0.808^*∗∗*^	0.224^*∗∗*^	0.580^*∗∗*^	0.242^*∗∗*^

^*∗∗*^Significant correlation at 0.01 level (bilateral), ^*∗*^Significant correlation at 0.05 level (bilateral). BMD: bone mineral density, E2: estradiol, T: testosterone, OPG: osteoporogeterin, RANKL: receptor activator of nuclear factor-*κ* B ligand, Cbfa-1: core-binding factor subunit alpha-1,COL-1: collagen?, OC: osteocalcin, BALP: bone alkaline phosphotase, TRACP-5b: tartrate-resistant acid phosphatase 5b.

## Data Availability

The data used to support the findings of this study are available from the corresponding author upon request.
